# Photoacoustic Imaging of Cancer Treatment Response: Early Detection of Therapeutic Effect from Thermosensitive Liposomes

**DOI:** 10.1371/journal.pone.0165345

**Published:** 2016-10-27

**Authors:** Jonathan P. May, Eno Hysi, Lauren A. Wirtzfeld, Elijus Undzys, Shyh-Dar Li, Michael C. Kolios

**Affiliations:** 1 Drug Discovery and Formulation Group, Drug Discovery Program, Ontario Institute for Cancer Research, Toronto, ON, Canada; 2 Department of Physics, Ryerson University, Toronto, ON, Canada; 3 Institute for Biomedical Engineering, Science and Technology, Keenan Research Centre for Biomedical Science, St. Michael’s Hospital, Toronto, ON, Canada; Brandeis University, UNITED STATES

## Abstract

Imaging methods capable of indicating the potential for success of an individualized treatment course, during or immediately following the treatment, could improve therapeutic outcomes. Temperature Sensitive Liposomes (TSLs) provide an effective way to deliver chemotherapeutics to a localized tumoral area heated to mild-hyperthermia (HT). The high drug levels reached in the tumor vasculature lead to increased tumor regression via the cascade of events during and immediately following treatment. For a TSL carrying doxorubicin (DOX) these include the rapid and intense exposure of endothelial cells to high drug concentrations, hemorrhage, blood coagulation and vascular shutdown. In this study, ultrasound-guided photoacoustic imaging was used to probe the changes to tumors following treatment with the TSL, HaT-DOX (Heat activated cytoToxic). Levels of oxygen saturation (sO_2_) were studied in a longitudinal manner, from 30 min pre-treatment to 7 days post-treatment. The efficacious treatments of HT-HaT-DOX were shown to induce a significant drop in sO_2_ (>10%) as early as 30 min post-treatment that led to tumor regression (in 90% of cases); HT-Saline and non-efficacious HT-HaT-DOX (10% of cases) treatments did not show any significant change in sO_2_ at these timepoints. The changes in sO_2_ were further corroborated with histological data, using the vascular and perfusion markers CD31 and FITC-lectin. These results allowed us to further surmise a plausible mechanism of the cellular events taking place in the TSL treated tumor regions over the first 24 hours post-treatment. The potential for using photoacoustic imaging to measure tumor sO_2_ as a surrogate prognostic marker for predicting therapeutic outcome with a TSL treatment is demonstrated.

## Introduction

During cancer treatment it is normal practice to monitor the tumor for changes indicative of treatment response. Conventionally, studies monitor volumetric changes in tumor size which typically occur weeks after the administration of treatment and thus are not suitable as markers of early treatment response [1]. Instead, dynamic or functional imaging techniques capable of monitoring the relative effectiveness of drug delivery [2–5], or better still detecting the corresponding therapeutic effect, during or immediately after treatment are highly sought after [6]. With this in mind, we recognized that many drugs and delivery therapies induce changes to the tumor microenviroment long before the overall volume visibly changes, and through further investigation it might be possible to use these for an early detection method of therapeutic effect.

**Fig 1:**
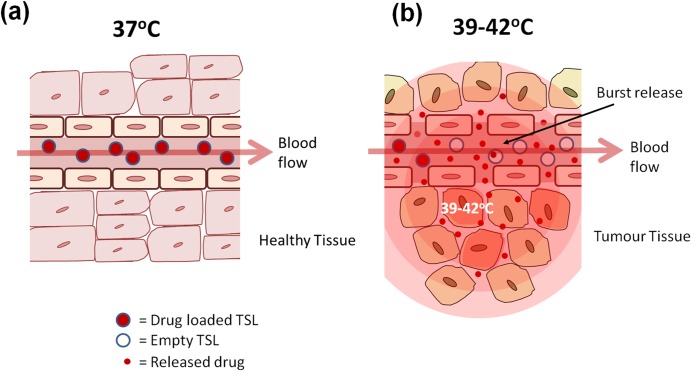
Schematic of the mode of action of a temperature sensitive liposome (TSL) for intravascular release. The TSL passes through normal unheated vasculature intact (a), but on reaching the heated tumor (b) drug is released in a burst-release fashion, creating a high local drug concentration which permeates into the tumor tissue.

TSLs are a drug delivery technology that allows the targeting of a drug payload to a localized area through the application of mild-hyperthermia (39–42°C) [7, 8]. The release temperature of a TSL can be tuned though the incorporation of lipids with different transition temperature (T_m_) or by adding other compounds (e.g. lyso-lipids, surfactants) to the lipid membrane. This approach has the potential to be particularly effective in cancer treatment, where heating (and so the drug release) can be confined to just the tumor area. This minimizes the uptake of drug elsewhere in the body and significantly reduces any unwanted side-effects associated with chemotherapy regimens [9]. A feature of the most clinically advanced ultra-fast temperature sensitive liposomes (uTSLs) is their ability to rapidly burst-release their drug payload (in seconds) when entering an area heated to mild-hyperthermia, but remain intact and retain the majority of their payload (for more than an hour) at normal physiological temperatures (Fig 1). Hence, mild-hyperthermia is usually applied within the first hour or two of uTSL treatment, for maximized concentration of encapsulated drug in circulation.

Lyso-lipid Temperature Sensitive Liposome (LTSL; DPPC/MSPC/DSPE-PEG, 86:10:4 mol%) is an example of such a burst-release TSL formulation [9, 10], which has progressed into late stage clinical trials delivering the drug doxorubicin (ThermoDox^®^, Celsion Corporation, Lawrenceville, NJ). This formulation is currently in clinical trials for hepatocellular carcinoma (phase III), for recurrent chest wall breast cancer (phase I/II) and for liver cancer (proof-of-principle study). Our group has previously reported on an improved TSL formulation, HaT (Heat-activated cytoToxic, DPPC:Brij78, 86:4 mol%), which exhibited increased release of doxorubicin (DOX) relative to LTSL (2-fold at 40°C and 1.2-fold greater at 41°C) [11–14]. In vivo, these release increments translated to improved tumour regression for HaT-DOX relative to LTSL-DOX.

The mechanism of action for an ultrafast TSL has been investigated and discussed in the literature for LTSL-DOX [15, 16]. These studies suggest that the mechanism and biological sequelae of events are different for an ultrafast TSL-DOX treatment compared to an infusion of free DOX, when each is combined with mild-hyperthermia. More pronounced activity is observed with the TSL treatment, where the rapid drug release leads to high levels of DOX in the vasculature. Subsequently, the drug attacks endothelial cells, and diffuses into the interstitial space, leading to tumor cell death. In such a case, the damaged endothelial cells may no longer offer the necessary support to contain the blood and its contents. This leads to vascular hemorrhage across the microvessel boundary, blood coagulation and vascular shutdown [11, 14]. These effects are more prominent for tumor vessels due to their inherent permeability, structural immaturity, and high proliferation in these regions of active angiogenesis [16]. Therefore, it is hypothesized that these events could be used as an endogenous physiological marker for an effective treatment and a potential surrogate marker of therapeutic effect. One such marker is the tumor blood oxygenation, and we sought to investigate this parameter in the context of relative levels of oxygen saturation (sO_2_) throughout the treatment period with HaT-DOX and mild-hyperthermia.

Longitudinal monitoring of blood sO_2_ as a function of treatment response requires an imaging modality which can non-invasively monitor oxygenation with sufficient spatial resolution and accessibility. Optical imaging techniques are capable of measuring oxygenation, but are severely limited in their penetration depth and spatial resolution due to the dominance of ballistic photon scattering [17]. Blood Oxygenation Level Dependent contrast (BOLD) MRI and oxygen-enhanced (OE) MRI are the only clinical imaging modalities capable of assessing volumetric tumour oxygenation [18–20]. However, these techniques are limited by their cost and accessibility which renders them impractical for assessing the early changes in oxygenation. Photoacoustic (PA) imaging has shown a great deal of promise in combining the most advantageous features of optical modalities (contrast) and ultrasound (US) technologies (resolution) [21–27]. Indeed, there are also recent reports correlating PA imaging data with that of the aforementioned MRI methods.[28, 29] PA images are acquired by detecting the ultrasonic pressure waves which are generated from the thermoelastic expansion of tissue as a result of short laser illumination. Sweeping of the optical wavelengths of illumination allows for functional PA imaging as selective absorption of the tissue chromophores would give rise to the multiple sources of contrast contained within the PA data. In the case of oxygenation, PA imaging has been able to compute absolute values of sO_2_ by taking advantage of the oxygen-dependent optical absorption of the hemoglobin (Hb) inside red blood cells [30]. Photoacoustic imaging has yielded a great deal of interest over the past few years, with its ability to provide co-registered structural and functional information on a wide variety of biomedical applications. However, most approaches have focused on engineering advances with the aim of improving the spatial resolution of the technique [31]. The application of PA imaging to the treatment monitoring problem has only begun recently with several encouraging studies demonstrating the potential of the technique for detecting changes of sO_2_ in the tumor vasculature as a function of treatment [32] as well as during tumor development [33] and even imaging vascular perfusion [34].

Our study utilizes PA imaging to provide new insights into understanding the mechanism of action of the HaT-DOX TSL formulation and investigates the feasibility of PA imaging for cancer treatment monitoring. We utilize PA imaging to map the changes in the oxygenation of multiple slices within a murine breast cancer model in the footpad treated with our TSL formulation, HaT-DOX. The efficacy of this treatment was studied relative to a saline control, and all treatments were combined with an application of mild-hyperthermia (HT). Very early changes in sO_2_ were examined for each tumour and these were correlated to the long term treatment outcomes.

## Materials and Methods

### Materials

1,2-Dipalmitoyl-sn-glycero-3-phosphatidylcholine (DPPC) was purchased from Avanti Polar Lipids (Alabaster, AL). Brij78 [(polyethyleneglycol-20) stearyl ether], sepharose CL-4B and FITC-lectin were bought from Sigma Aldrich (Oakville, ON, Canada). DOX was purchased from Tocris Bioscience (Ellisville, MO). All other reagents were of analytical grade.

### Preparation of HaT-DOX liposomes

HaT liposomes were prepared by thin lipid-film hydration followed by membrane extrusion to control size, as described in previous reports [11, 12]. Briefly, 45 mg of lipids (DPPC/Brij, 96:4 mol%) were dissolved in isopropanol and the solvent was evaporated under a flow of nitrogen gas at ~60°C. The resultant lipid film was dried further under high vacuum overnight to remove any residual organic solvent. Lipid films were hydrated with 300 mM citric acid (1 mL) to form multilamellar vesicles, which were then extruded 21 times through polycarbonate filters (pore size: 0.1 μm) at 65°C to adjust the liposome size. Following extrusion, formulations were cooled to room temperature and checked for size and polydispersity index (PDI) by dynamic light scattering (DLS).

To load DOX into the liposomes, a pH gradient was used to obtain a high loading of drug via a remote loading strategy. This method concentrates the drug into the liposome core through the protonation and trapping of DOX upon entering the liposome core. The pH gradient was generated by first exchanging the exterior buffer with HBS (25 mM HEPES buffered saline, pH 7.4) via dialysis (Slide-A-lyzer 10 kDa MWCO, Pierce Biotechnology, Rockford, IL). The dialysis buffer (500 mL) was exchanged every hour for 3 hours, at which point the pH was checked to ensure it was close to neutral. The liposome and DOX were then incubated at 37°C for 90 min at a 20:1 ratio (w/w), respectively. Following incubation, the un-encapsulated DOX was removed by purification with a sepharose CL-4B column eluting with HBS. The liposome fraction was analyzed for any change in size, PDI and drug content. DOX concentration was determined using fluorescence (excitation: 485 nm; emission: 590 nm), before and after liposome membrane disruption (Triton X-100) using a fluorescence plate reader (Hidex, Finland) as described previously [11, 12]. Particle size distributions were measured by dynamic light scattering (Zetasizer Nano-ZS, Malvern Instruments Ltd, UK). All experiments were performed with freshly prepared formulations.

### Cell culture and animal models

The murine breast cancer cell line EMT-6 was purchased from ATCC (Manassas, VA). EMT-6 cells were maintained in DMEM supplemented with 10% FBS, penicillin (100 U/mL) and streptomycin (100 μg/mL) at 37°C with 5% CO_2_. Female BALB/c mice (aged 5–6 weeks, 18–20 g) were purchased from Harlan (Mississauga, ON, Canada). All experimental protocols in this study were approved by the Animal Care Committee of the University Health Network (Toronto, ON, Canada) in accordance with the policies established in the Guide to the Care and Use of Experimental Animals prepared by the Canadian Council of Animal Care. Mice were housed in individually ventilated cages (up to 5 mice per cage) supplied with acidified automatic watering system. Teklad irradiated rodent diet #7912 ad lib, autoclaved corn cob bedding or iso-PADS bedding was used to minimize agitation of tumors. Every cage provided with autoclaved enrichment (a translucent, red polycarbonate house and nestlets for nest building). The animal room operates at 20–22°C, 40–70% relative humidity, with a light/dark cycle of 12/12 hr. All animals were sacrificed with Isoflurane anesthetic followed by CO_2_ asphyxiation. If any signs of pain or suffering were observed then analgesics were applied. Animals were under veterinary observation on a routine basis.

### In vivo treatment protocol

The murine breast cancer cell line, EMT-6, was inoculated (1 × 10^6^ cells/50 μL medium) subcutaneously into the footpad of BALB/c mice. The footpad thickness was monitored, and after ∼7 days a measurable change in thickness (1.0–2.0 mm) was observed due to tumor growth. At this point, the mice were deemed ready to undergo the treatment/imaging protocol as depicted in S1 Fig. Following the initial pre-treatment image, each mouse was treated with one of the formulations: HaT-DOX (10 mg DOX/kg, n = 13) or Saline (HBS pH 7.4, n = 15), via intravenous tail vein injection. This was immediately followed by localized heating of the tumor-bearing hind limb footpad with a water bath at 43°C for 1 h (S1A Fig). This temperature and time period was determined to be optimal for maintaining the tumor in the mild-hyperthermia range in previous studies [11–14]. During the treatment period, mice were anesthetized with a flow of isofluorane (1.5%) in oxygen (0.5–1 L/min). Following treatment the mice were returned to their cage and monitored closely to ensure full recovery and taken for imaging at further timepoints as described below. Mice were monitored regularly (every 1–2 days) for changes in footpad thickness (measured by standard calipers) and body weight. Mice were euthanized when the tumors reached double their original size (original size = treatment day = day 0) in a single dimension, or reached endpoint via some other means (e.g. open tumor, 20% body weight loss, immobility etc.). If a treatment showed a reduction in tumor size at endpoint relative to the tumor’s original size at day 0, this was defined as regression. Regression rate for a particular group was defined as the number of mice that showed regression divided by the total number of mice for each particular group.

### In vivo imaging protocol

Imaging of each animal was performed with the Vevo LAZR US/PA small animal imaging device (Fujifilm VisualSonics Inc., ON, Canada). This is a commercial system that consists of a 256-element, 40 MHz center frequency, linear array US/PA probe coupled to an Nd:YAG laser operated through an optical parametric oscillator with a 6 ns pulse length, 20 Hz pulse repetition frequency and 680–970 nm output. For the purposes of this study, the tumors were independently illuminated with 750 and 850 nm wavelengths. These two wavelengths were chosen to probe the optical properties of blood either side of the isosbestic point (805 nm, the optical wavelength at which the absorption of oxygenated and deoxygenated blood is the same). During imaging, all mice were anesthetized with a flow of isofluorane (1.5%) in oxygen (0.5–1 L/min). Clear ultrasonic gel was used to acoustically couple the footpad of each animal with the imaging probe, while the core body temperature was maintained at ~37°C using a heating platform (S1B Fig). Co-registered, 3D US and PA images were acquired by scanning the imaging probe over the entire tumor volume (81 frames, 80 μm apart).

Imaging was performed at set timepoints before and after treatment (S1C Fig). A pre-treatment image was taken 30 min prior to treatment for each animal. Each animal then received its dose of formulation and was immediately placed in the water bath heating set-up to receive localized mild-hyperthermia (HT) to the tumor bearing hind limb as described above. Following treatment, the animal was imaged at 5 further timepoints: at 30 min, 2 h, 5 h, 24 h and 7 days post-treatment.

### Ultrasound and photoacoustic imaging and data processing

At each imaging timepoint, a total of 21 US/PA, 2D B-mode frames (80 μm apart) were analyzed (10 on either side of the anatomical center of the tumor determined from the US B-mode image). Each 2D US image was used to anatomically segment the tumor in each frame, while avoiding the skin, bone and artifacts. The same region of interest (ROI) was applied to segment the PA images acquired at 750/850 nm for all of the 21 frames. The energy of each pulse at the two wavelengths was measured in real-time using an energy meter (Ophir-Spiricon, North Logan, Utah, USA) that was coupled to the image acquisition sequence.The PA images at each wavelength were normalized by their respective, real-time energies in order to remove the wavelength-dependent laser energy variations present within the system. The PA pressure in tissue is directly proportional to the absorbed energy in tissue with the same constant of proportionality (Grüneisen parameter) throughout tissue. Given that the tumors were small and superficial, no corrections were made for the differences in tissue optical fluence for the two wavelengths. Oxygen saturation (sO_2_) maps were generated by measuring the PA signal at 750/850 nm for each pixel within the tumor ROI. The sO_2_ was calculated based on the underlying assumption that the PA signal at the two wavelengths is primarily dominated by the optical absorption of hemoglobin (Hb) in its oxygenated (*μ*_*HbO*_) and deoxygenated (*μ*_*Hb*_) forms. Eq (1) shows the derivation of the sO_2_ from the relationship between optical absorption and chromophore concentration (oxygenated hemoglobin[*HbO*], or deoxygenated hemoglobin[*Hb*]) [35, 36],

$$\begin{array}{l} PA_{SA}\left(\lambda_{1}\right)\propto \mu_{a}\left(\lambda_{1}\right)=\left[Hb\right]\varepsilon_{Hb}\left(\lambda_{1}\right)+\left[HbO\right]\varepsilon_{HbO}\left(\lambda_{1}\right)\\ PA_{SA}\left(\lambda_{1}\right)\propto \mu_{a}\left(\lambda_{2}\right)=\left[Hb\right]\varepsilon_{Hb}\left(\lambda_{2}\right)+\left[HbO\right]\varepsilon_{HbO}\left(\lambda_{2}\right)\\ sO_{2}=\frac{\left[HbO\right]}{\left[HbO+Hb\right]}=\frac{PA_{SA}\left(\lambda_{2}\right)\times \varepsilon_{Hb}\left(\lambda_{1}\right)-PA_{SA}\left(\lambda_{1}\right)\times \varepsilon_{Hb}\left(\lambda_{2}\right)}{PA_{SA}\left(\lambda_{1}\right)\times \Delta \varepsilon \left(\lambda_{2}\right)-PA_{SA}\left(\lambda_{2}\right)\times \Delta \varepsilon \left(\lambda_{1}\right)}\\ \Delta \varepsilon \left(\lambda \right)=\varepsilon_{HbO}\left(\lambda \right)-\varepsilon_{Hb}\left(\lambda \right) \end{array}$$ (1)

where, *μ*_*a*_ is the optical absorption coefficient, *PA*_*SA*_(*λ*) is the photoacoustic signal amplitude at a particular wavelength of illumination (λ), calculated as the envelope of the time-domain PA signal within the region of interest; ε_Hb_ and ε_HbO_ are the extinction co-efficients of deoxygenated and oxygenated hemoglobin, respectively; Δ*ε* represents the difference in extinction coefficient between the oxygenated and deoxygenated hemoglobin. The wavelengths *λ*_1_ and *λ*_2_ correspond to 750 and 850 nm, respectively.

A schematic of the algorithm used to compute the sO_2_ maps and histograms is shown in S2 Fig. In order to quantify the sO_2_ distribution of each tumor slice, a novel approach was employed where the sO_2_ intensity of all pixels within a given frame was represented in the form of a histogram. At each imaging timepoint, for each mouse, the average of 21 histograms was computed along with the standard deviation of the pixel count of each sO_2_ value. The resultant plot represents the temporal change in sO_2_ for each tumor, which were used to quantify the changes in tumor sO_2_ as a function of time and treatment type. This approach allows for quantification of the tumor oxygenation without relying on image processing algorithms that might affect the estimated sO_2_. All image and signal processing was performed in Matlab2014a (The MathWorks Inc., Natick, MA).

### Tumor histology

For each treatment, at least 6 mice were used to study tumor histology at two key timepoints; 2 h and 7 days post-treatment. These mice were randomly pre-selected for FITC-imaging prior to treatment with 3 mice being used for each timepoint of a particular treatment. Timepoints were chosen to represent both a very early timepoint post-treatment and a timepoint sufficiently late enough to begin to observe the early signs of treatment efficacy via conventional tumor measurement methods. Animals used for tumor histology were injected intravenously with FITC-lectin (0.25 mg/mL, 200 μL) following their final US/PA image and returned to their cage. After 1 h, mice were euthanized and their footpad tumors were removed and cryogenically frozen in Optimal Cutting Temperature (OCT) gel for sectioning, staining and processing by the Pathology department at the STTARR facility (Toronto, ON, Canada). Sections were stained with H&E; fluorescent immunohistochemical stains for the vascular marker, CD31; and cell nuclei marker, DAPI. The sections were then scanned to identify the presence of FITC-lectin (green channel), cyanine dye labelled CD31 Ab (red channel) and DAPI (blue channel). Images were then processed using the Definiens software package (Munich, Germany) to quantify the intensity and distribution of each stain.

### Statistical analysis

All data are expressed as mean ± standard deviation (S.D.). Statistical analysis was conducted with the two-tailed unpaired *t test* for two-group comparison, or one-way ANOVA, followed by the Tukey multiple comparison test by using GraphPad Prism (for three or more groups). A *p*-value of less than 0.05 was considered to be statistically significant.

## Results

### Characterization of HaT-DOX TSLs

The HaT-DOX liposomes were prepared as described in our earlier publications [11–13]. These liposomes were studied for size and drug loading to ensure all batches of liposomes possessed comparable physical characteristics (S1 Table). In all cases, the size observed by dynamic light scattering was within 90–100 nm with a PDI of ~0.06. Drug loading efficiency was generally very high (~100%) for the remote loading method used, and final drug loaded liposome concentrations were adjusted to 1 mg/mL with a drug-to-lipid ratio of ~0.05 (w/w).

### Tumor efficacy for a murine footpad model

**Fig 2:**
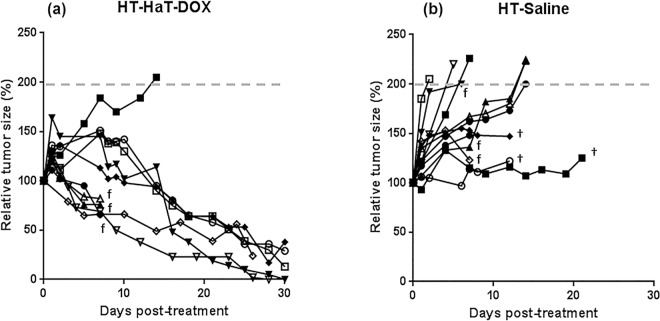
**Tumor growth plots for mice treated with 1 hour of hyperthermia (HT) and an intravenous dose of (a) HaT-DOX (n = 10) or (b) Saline (n = 12).** Tumors were grown subcutaneously in the footpad of the right hind limb and changes in size were measured regularly with calipers. HT-HaT-DOX treatments were dosed at 10 mg DOX/kg. The dashed line (——) represents the endpoint due to tumor load. The **†** symbol indicates mice which reached a premature endpoint due to tumor ulceration or lack of sufficient mobility. The **f** symbol indicates all mice that were sacrificed for histology at 7 days post-treatment.

Previous studies have demonstrated improved efficacy with the HaT-DOX treatment relative to LTSL-DOX or DOX [11–14]. In preliminary studies, the DOX fluorescence of EMT-6 tumors was measured after treatment with equal doses (10 mg DOX/kg) of each of these 3 treatments (S3 Fig). HaT-DOX showed considerably greater tumoral drug uptake, and on this evidence, together with work from previous studies, [11–14] HaT-DOX was chosen as the TSL for further study in this work. The therapeutic effect of the HaT-DOX (10 mg DOX/kg) and Saline control (sterile HBS pH 7.4) formulations were studied in a subcutaneous footpad EMT-6 tumor model with mild-hyperthermia (HT) for a period of 1 h. Tumor size and animal body weights were then followed to assess the relative efficacy of each treatment. All treatments that show a reduction in tumor size at endpoint relative to day 0 were classified as showing regression. The relative tumor sizes (%) over the study period were plotted for each animal (Fig 2). It is worth mentioning that many treatments underwent a transient inflammation and swelling of the treated area for a few days post-treatment–this was particularly noticeable for many of the HT-HaT-DOX treated mice (Fig 2A**)**.

The HT-Saline treated mice may also have shown some inflammation although it was more challenging to differentiate this from tumor growth. Although there was some natural variation with each treatment, it can be seen that HT-HaT-DOX treatments generally resulted in good tumor regression (9/10 mice showed regression over 25 days), whereas HT-Saline treatments (Fig 2B) showed no regression, and the majority of these tumors reached endpoint by 14 days post-treatment (tumor size 200% relative to the size on day 0). The trends are consistent with results reported previously by our group and others for TSL and buffer control treatments combined with mild-hyperthermia [8, 12]. None of the treatments demonstrated signs of significant toxicity as represented by each of the animal’s changes in body weight (<10% variation) during the treatment/imaging course (S4 Fig).

### Longitudinal mapping of tumor sO_2_

In previously unpublished preliminary work (S5 Fig), the HT-HaT-DOX treatment was studied with a window chamber tumor model indicating what appeared to be localized hemorrhage and bleeding in the vicinity of the tumor. This not only highlighted to us an interest in imaging a blood dependent parameter, but also provided guidance for the selection of the appropriate timepoints for the subsequent study. Hence, the HT-HaT-DOX and HT-Saline treated animals in this study were imaged at several timepoints using non-invasive, co-registered ultrasound and photoacoustic methods in order to test the hypothesis that sO_2_ might be a surrogate prognostic marker for effective HT-HaT-DOX treatment.

**Fig 3:**
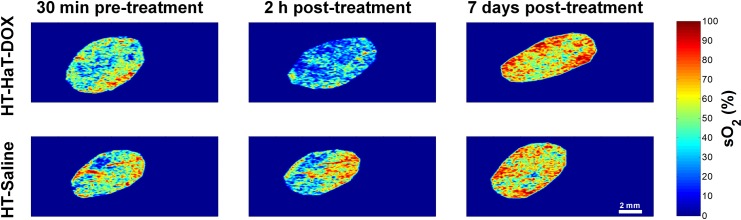
Representative sO_2_ maps, These are shown for HT-HaT-DOX (top row) and HT-Saline (bottom row) treated tumors at 30 min pre-treatment (1^st^ column), 2 h post-treatment (2^nd^ column) and 7 days post-treatment (3^rd^ column). The scale bar (2 mm) and sO_2_ color bar (0–100%) apply to all sO_2_ maps shown.

The sO_2_ maps derived from two-wavelength photoacoustic imaging of tumors represent the relative spatial distribution of oxygen saturation within the tumor (S6 Fig). A time-dependent change of sO_2_ was observed for the HT-HaT-DOX group and to demonstrate this visually the overall trend of the group can be represented nicely in just a few imaging timepoints from one of these animals (Fig 3). Each 2D map denotes the sO_2_ of blood inside the segmented tumor ROI at 30 min pre-treatment, and 2 h and 7 days post-treatment. For HT-HaT-DOX treated mice, a significant drop in the tumor sO_2_ was observed at 2 h post-treatment compared to the 30 min pre-treatment image. At 2 h post-treatment, while some blood within the tumor still contained moderate to high sO_2_ values (orange/red colour in Fig 3), the majority of the tumor exhibited very low sO_2_ (blue colour in Fig 3). It is important to note that the significant drop in the tumor sO_2_ for the HT-HaT-DOX treated mice was apparent as early as 30 min post-treatment and it remained at these levels for more than 5 h (S6 Fig).

The HT-Saline group received injections of HBS (pH 7.4) prior to undergoing an identical mild-hyperthermia treatment protocol to the other animals that received drug formulation. The sO_2_ for the HT-Saline group did not show the decrease in sO_2_ observed for the HT-HaT-DOX mice at 2 h post-treatment (Fig 3, bottom row). For the specific HT-Saline treated mouse represented in this figure, the sO_2_ shows a slight increase from the 30 min pre-treatment timepoint to the 2 h post-treatment timepoint image, as represented qualitatively by the increase in red and decrease in blue colour in the image.

The trends represented in Fig 3 were consistent across each respective group, i.e. HT-HaT-DOX treatment led to an immediate drop in tumor sO_2_ which was sustained for at least the first 5 h post-treatment, while for HT-Saline no such drop in sO_2_ was observed and levels remained relatively constant for the same period with minor fluctuations (S6 Fig). Upon reaching the 7 day timepoint both groups exhibited a similar behaviour, where the overall sO_2_ rose above the level observed for their respective 30 min pre-treatment images.

#### Oxygen saturation (sO_2_) histograms and quantification of the changes in oxygenation

**Fig 4:**
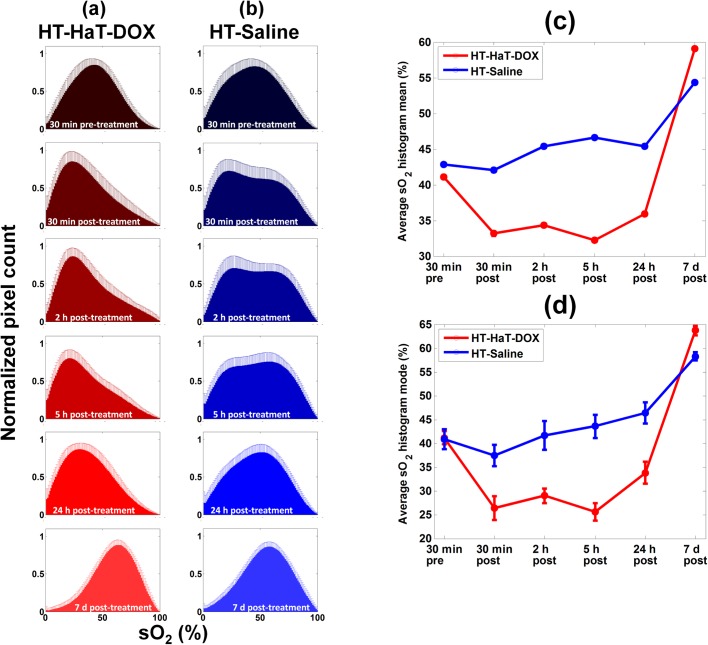
Oxygenation histograms. (a) Average sO_2_ histograms at 30 min pre-treatment and 30 min, 2 h, 5 h, 24 h and 7 day timepoints post-treatment for HT-HaT-DOX (n = 12) and HT-Saline (n = 15). Two timecourses have been plotted to compare the treatments studied using the mode (b) and mean (c) averages of the histogram data plotted in (a) relative to their starting values at 30 min pre-treatment. Error bars represent the standard deviation on the pixel count for each sO_2_ value from each mouse which had 21 different histograms per imaging timepoint. The black arrows represent the points at which the treatments were made (i.e. defined in this plot as 0 h). Datapoints that show a drop in sO_2_ which is significantly different to pre-treatment are represented by * where p < 0.05.

In order to quantify the relative sO_2_ of the blood in tumors and capture the heterogeneity of the entire tumor volume, histograms of the distribution of sO_2_ values (number of pixels with a certain sO_2_ value as a function of that sO_2_ value) were calculated (Fig 4). The histogram of every imaging slice was combined to create an average histogram representing a treatment group at a given timepoint (30 min pre-treatment and at 30 min, 2 h, 5 h, 24 h and 7 days post-treatment, S2 Fig).

Regardless of the timepoint or treatment type, all histograms showed a distribution of sO_2_ values. The histogram of the HT-HaT-DOX treated animals revealed a significant shift to the left during the first few timepoints, representing a drop (>15% in histogram mode) in oxygen saturation (Fig 4A). This shift was apparent as early as 30 min post-treatment and it persisted for the first 5 h, in agreement with the sO_2_ map images (Fig 3 and S6 Fig). The histogram shifted to higher sO_2_, approaching that of pre-treatment levels by 24 h, and by 7 days it had surpassed the values of the pre-treatment histogram. This dynamic shift in the distribution of blood sO_2_ values within the tumor was quantitatively represented by the early drop (30 min to 5 h post-treatment) and late (7 day) increase in tumor sO_2_ observed following HT-HaT-DOX treatment.

Histograms for the HT-Saline group exhibited comparable 30 min pre-treatment sO_2_ distributions to the HT-HaT-DOX group (modes ~ 40%). However, there was no significant drop in the average sO_2_ (either mean or mode) during the first 5 h post-treatment, and again an increase was observed by 7 days post-treatment. In general, the post-treatment histograms of the HT-Saline group appeared to have broader distributions and displayed increased bimodal character (e.g. 2 h histogram for HT-Saline in Fig 4A), than observed for the HT-HaT-DOX group.

Given that a distribution of sO_2_ values exists within the tumor, one must quantify the changes in the sO_2_ over time by focusing on the statistics of the histograms. The mode of the sO_2_ within the tumor, represented by the peak of a histogram, can be representive of how the sO_2_ distribution varies over time, and this is particularly effective for HT-HaT-DOX, but this is less meaningful for the broad distributions for the HT-Saline treatment, particularly when bimodal character is observed. For this reason, we studied both the mode and mean sO_2_ change over time (Fig 4B and 4C). For the HT-HaT-DOX treated mice, a significant drop in the mean (~10%) and mode (~15%) of the histogram was observed from 30 min pre-treatment to 30 min post-treatment. This is indicative of an overall shift of the entire tumor region to reduced sO_2_ after treatment, and is visually represented as a shift of the entire histogram distribution to the left (Fig 4A). The drop in the mean sO_2_ value was sustained for the first 5 h. Following this initial period, a gradual increase in mean and mode sO_2_ for HT-HaT-DOX was observed until day 7, reaching levels 15–20% above those at 30 min pre-treatment.

The changes in mode and mean of the sO_2_ histogram for the HT-Saline group (Fig 4B and 4C) were quite different to those of the HT-HaT-DOX group over time. The histogram mode showed very little change from pre-treatment during the first 5 h post-treatment. The mean sO_2_ also varied much less than for HT-HaT-DOX but showed a slight increase (2–3%) in sO_2_ over the first 5 h post-treatment relative to pre-treatment. By the 7 day timepoint, the mean sO_2_ had increased to ~10% above pre-treatment levels.

### Correlation between early changes in sO_2_ and treatment efficacy

**Fig 5:**
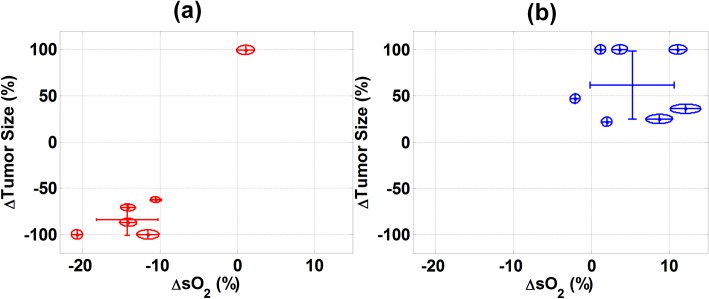
Size and oxygenation relationships. Correlation between the changes in the size of the tumor treated with (a) HT-HaT-DOX and (b) HT-Saline at endpoint (from day 0) and the changes in mean sO_2_ between the values observed for 30 min pre-treatment and 2 h post-treatment. Each point is the average of 21 sO_2_ histograms at the 2 h timepoint. The major and minor axes of each ellipse represent the standard deviations of the change in sO_2_ and change in tumor size, respectively. † identifies a datapoint for a HT-HaT-DOX treatment that did not show regression, nor a characteristic drop in sO_2_ at 2 h post-treatment.

The relationship between the change in tumor size (at endpoint relative to day 0) and the change in the mean sO_2_ (at 2 h post-treatment relative to 30 min pre-treatment) was plotted for the mice of both treatment groups (Fig 5**)**. From analysis of the HT-HaT-DOX group there was a significant separation between the mice which showed regression and the one that did not. The mice that responded to treatment exhibited a decrease in tumor size of at least 50% by their endpoint and their mean sO_2_ at 2 h had dropped by an average of 10–15% from 30 min pre-treatment values. This figure demonstrates how a large drop (>10%) in mean sO_2_ at 2 h post-treatment was typically correlated with a large tumor regression by endpoint. The majority (90%) of animals from the HT-HaT-DOX group are contained within one standard deviation of the mean. The HT-Saline treated mice did not show such a clear trend as represented by the wide distribution of data points and larger standard deviation on both axes.

A single HT-HaT-DOX treated mouse (marked † in Fig 5) did not show the characteristic drop in sO_2_ of more than 10% by 2 h post-treatment as observed for the other HT-HaT-DOX treated animals. In fact, this treatment displayed no significant decrease in sO_2_ throughout the first 5 h post-treatment. Furthermore, no regression was observed for this particular treatment, with the tumor increasing in size to >200% in just 7 days.

### Histological analysis of tumor treatment

To investigate the cause for the observed changes in sO_2_, a second experiment was performed where animals were sacrificed at two distinct timepoints post-treatment (2 h and 7 days) following treatment with mild-hyperthemia and either HaT-DOX or Saline. These timepoints were chosen in order to represent the key changes observed with PA imaging.

**Fig 6:**
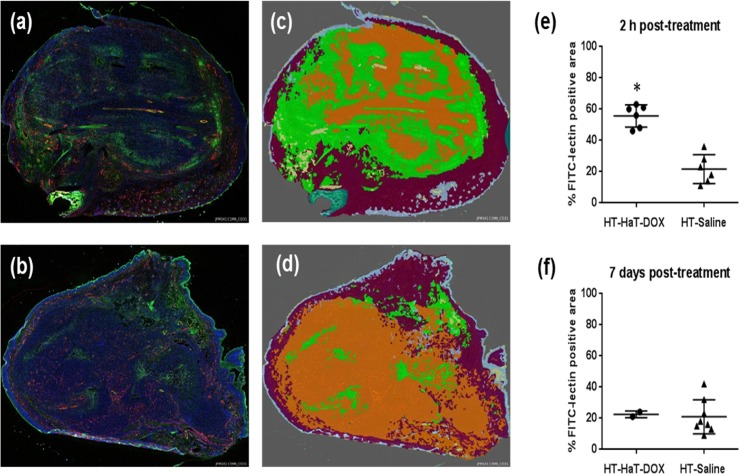
Tumor histology. Representative sections of footpad tumor harvested at the 2 h timepoint and stained with CD31 (red), DAPI (blue) and FITC-lectin (green) for (a) HT-HaT-DOX, and (b) HT-Saline treated mice. The same sections displaying the regions defined as FITC-positive tumor (green), FITC-negative tumor (orange) and normal tissue (maroon), after processing with Definiens software for (c) HT-HaT-DOX and (d) HT-Saline. Column scatter plots: Relative FITC-postive areas following quantification with a Definiens analysis at (e) 2 h and (f) 7 days post-treatment; Significance is represented by * where p < 0.0005.

Sections of HT-HaT-DOX treated tumor showed significant FITC-lectin perfusion and leakage from the vasculature at the 2 h timepoint (Fig 6A and 6C). This level of FITC leakage was not observed for the HT-Saline group at 2 h or 7 days (Fig 6B and 6D), nor for the HT-HaT-DOX treated mice at 7 days post-treatment. The area of FITC positive tumor was analyzed with Definiens software to provide quantitative results (Fig 6E and 6F), demonstrating an average area of ~ 60% FITC positive tumor for the HT-HaT-DOX mice at 2 h, while only 20–40% was observed for all timepoints with the HT-Saline treated animals. The level of vessel perfusion was also studied and showed a similar trend between treatments and timepoints (S7 Fig). This data also correlates well with observations using the window chamber model (S5 Fig) with significant FITC-leakage/bleeding observed in both cases at the 2 h post-treatment timepoint.

## Discussion

There is a need for chemotherapies that are localized in their action; this could provide a means of limiting toxicity to normal tissue and improve the therapeutic window of the drugs involved [37]. Triggered release nanoparticles provide one way this could be achieved [38–40], an example of which are TSLs [7]. Regardless of the treatment type, emerging evidence suggests that early assessment of therapeutic effect has the potential to have a significant clinical impact [17, 41–45]. The early readout of treatment efficacy could potentially be achieved directly through measurements of relative levels of endogenous biomarkers [46, 47]; an approach which is warranted by the complex nature of cancer growth and treatment response that requires personalized therapies as well as personalized means of assessing treatment outcome [47]. Even with the impressive advancements in personalized medicine and nanotechnology, current practice for assessment of cancer treatment efficacy is often limited to the anatomical information obtained through imaging studies using magnetic resonance or computed tomography. These methods can be prohibitively costly, often require the use of contrast agents with lengthy scan times, and most commonly measure the change in tumor size which may not be apparent until weeks after treatment. The motivation for this study stemmed from the desire to assess therapeutic effect shortly after treatment (< 6 h) in order to make reasonable estimates of treatment prognosis and potential success on a personalized level.

Here, we have studied a TSL developed in our lab (HaT-DOX), designed to release its payload at mild-hyperthermia (HT, 39–42°C), and probed the tumor region in vivo with US-guided PA imaging throughout the course of the treatment period (from 30 min pre-treatment to 7 days post-treatment). We sought to demonstrate improved treatment efficacy with the HaT-DOX formulation over that of Saline when each was combined with mild-hyperthermia. In addition to demonstrating therapeutic effect, we also investigated the structural and functional changes taking place within the tumor during and following treatment, by using the non-invasive methods of ultrasound (US) and photoacoustics (PA).

HaT-DOX TSLs were prepared in a similar manner to that described previously [12, 13], and studied with a tumour footpad model, allowing the application of mild-hyperthermia (43°C) localized to just the tumor-bearing hind limb. In doing so, drug delivery was targeted to the tumor region and compared with Saline (HBS pH 7.4) control using the same mild-hyperthermia heating method (HT). HT-HaT-DOX showed good efficacy with 90% of tumors demonstrating significant regression by endpoint. Meanwhile the HT-Saline treatment was essentially ineffective, with no treatments displaying regression. These data closely match responses observed in previous reports with this formulation [12] and other related intravascular “burst-release” TSLs [48]. It is worth noting that while tumor inflammation most likely influenced absolute measurements of tumor volume in the first 3–10 days post-treatment, but this had no affect on the relative change at the study endpoint necessary to classify tumor response.

After demonstrating the differences in therapeutic effect, our study investigated the potential of US-guided PA imaging for non-invasive cancer treatement monitoring. PA imaging is relatively new to this field, but it offers a great deal of promise in being able to provide co-registered functional and structural information without any endogenous contrast [33, 49]. Within the resolution limits of our imaging system (45 μm axial, 90 μm lateral), PA imaging was capable of capturing the oxygenation of the blood up to a depth of 11 mm at 40 MHz. At lower frequencies, PA has even been shown to map the location of vessels as deep as 40 mm in breast tissue [50]. The added spatial resolution at clinically relevant depths yields a distinct advantage of PA imaging over other optical methods that are limited to sub-micron depths due to ballistic photon scattering [25].

Based on preliminary window chamber data (S5 Fig), prior studies, and the proposed TSL mechanism of action (vide supra), it was hypothesized that the drug induced damage to the vasculature and tumor tissue could lead to the entrapment of deoxygenated red blood cells (known as blood pooling) in the perivascular space of the HaT-DOX treated tumors. These deoxygenated red blood cells would likely remain in this state until injury repair mechanisms start to regenerate the treatment area. Hence, during this period it should be possible to observe a distinct drop in oxygen saturation (sO_2_) for all successful TSL treatments and this would be detectable with non-invasive PA imaging. Therefore, imaging timepoints ranging from 30 minutes to 7 days post-treatment were chosen based on the preliminary window chamber data collected, and the prior work on the mechanism of TSL drug action on the tumor vasculature [8].

The potential for using the change in the sO_2_ as a surrogate marker for therapeutic effect following TSL treatment was investigated. Following treatment with the TSL HaT-DOX, we found a strong correlation between the early changes in the sO_2_ of a tumor, and the change in tumor volume in the longer-term. While the sO_2_ maps for both the HT-HaT-DOX and HT-Saline treated mice appeared very similar at 30 min pre-treatment, there was a clear change between the two groups at the 30 min post-treatment timepoint. At this early timepoint, the mode of the sO_2_ values was seen to drop significantly (~10–20%) for the HT-HaT-DOX treated mice, but not for the HT-Saline group; an effect that lasted for up to 5 h post-treatment. The drop in sO_2_ for the HT-HaT-DOX treatment correlated well with a significant tumor regression (90% regression rate) 28 days post-treatment. No such drop in sO_2_ or tumor regression was observed for the HT-Saline treatment, indicating the significance of the HaT-DOX component. To the best of our knowledge this is the first time an imaging modality has been used to study the effect of a TSL on the tumor environment in order to predict the long term therapeutic outcome.

**Fig 7:**
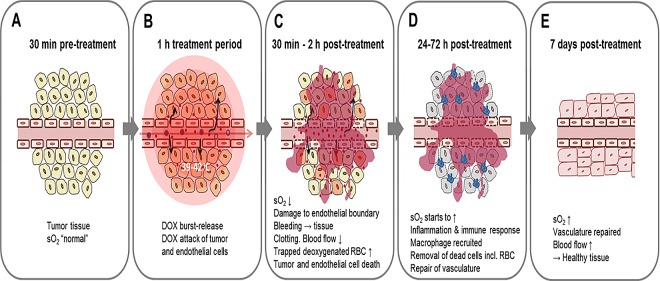
Proposed mechanism and levels of oxygen saturation (sO_2_) following treatment with a burst-release TSL such as HaT-DOX triggered with mild-hyperthermia. The timecourse is represented as a number of snapshots which appear sequentially from A-E.

The proposed mechanism for an ultrafast burst-release TSL (such as HaT-DOX) provides sufficient information to account for the significant drop in sO_2_ observed for the HT-HaT-DOX treated tumors between 30 min to 5 h post-treatment, as well as agreeing with the histological data obtained. Once vessels are disrupted due to the physiological effects of the HT-HaT-DOX treatment (likely more pronounced for the neo-vascularture of the tumor region), their ability to circulate oxygenated red blood cells is diminished, resulting in a drop of tumor sO_2_ levels (Fig 7). The high concentrations of DOX released inside the tumor vasculature leads to damage of both endothelial and tumor cells as the drug rapidly permeates out of the vessels and into the surrounding tissue, aided by the damaged vasculature as demonstrated by the increased levels of FITC-lectin observed for these tumors (Fig 6) and significant hemorrhage within the tumor (as observed in the preliminary window chamber study (S5 Fig). The observed low sO_2_ environment persists for more than 5 h, after which a gradual increase in tumoral sO_2_ starts to be observed. During this time, the natural repair mechanisms of the body will be prevalent, leading to recruitment of macrophages and immune cells for clean-up and regeneration of the damaged vasculature and surrounding tissue [51]. This was observed as a transient inflammation and swelling of the treated area for the first few days post-treatment. Approximately three to ten days later this inflammation had begun to subside and it appeared the vasculature had been repaired, accounting for the increase in sO_2_ observed at 7 days. These findings are further supported by the relatively small changes in tumor size over the first week for the HT-HaT-DOX group, which could also be explained by such inflammation and tissue regeneration.

Unlike the HT-HaT-DOX group, the HT-Saline group showed no regression, very little change in sO_2_ (30 min to 5 h post-treatment, Fig 4) and in most cases a significant increase in tumor size was observed. However, much like the HT-HaT-DOX group, by 7 days the HT-Saline group also showed a significant increase in sO_2_. In this case we speculate that the increase is due to the recruitment of new vessels required to maintain tumor growth. Consequently, the key difference observed in this study between the HT-HaT-DOX and HT-Saline groups remains the drop in sO_2_ observed for HT-HaT-DOX within the first 5 hours after treatment, which corresponded to the desired therapeutic effect. In future work, it would be of interest to further investigate the mechanism of action by computing the total hemoglobin concentration as a function of time. Utilizing spectral unmixing approaches[52] might elucidate the upconversion of oxyhemoglobin to its deoxygenated counterpart and provide further evidence of vascular shutdown due to HT-HaT-DOX.

From studying the distribution of results expressed as change in tumor size (day 0 to endpoint) versus change in mean sO_2_ (30 min pre-treatment to 5 h post-treatement) a clear segregation between efficacious and non-efficacious treatments was observed. We propose that with further work a threshold sO_2_ drop could be identified, which would represent the “cut-off” drop in sO_2_ required for a treatment to show significant tumor regression. For example, in the current study a single HT-HaT-DOX treated animal may have been identified as an ineffective treatment; not reaching a threshold drop in sO_2_ and also not demonstrating a characteristic tumor regression response (Figs 2 and 5). Treatment response is affected by a wide array of factors that stem from ineffective delivery of the targeted therapeutic payload, to biological variability in the tumor’s biology [53]. In the case of this HT-HaT-DOX treated animal, the tumor grew to 200% of its original size in 2 weeks, while the mean change of the sO_2_ histogram mean between 30 min and 2 h post-treatment was not significantly different to values recorded pre-treatment (*p* not less than 0.05). It is encouraging that this HT-HaT-DOX treated animal, which showed no regression, also did not reach the threshold sO_2_ that this work suggests is necessary for a therapeutic response. This reinforces our hypothesis that sO_2_ has the potential to be used as a predictor of therapeutic effect for ultrafast TSL treatments like HaT-DOX. The results of this study suggest that there is indeed added value to probing the tumor sO_2_ at depths that are clinically meaningful and may not be reached by means other than PA imaging.

As PA imaging begins to make its transition into the treatment monitoring arsenal, it is encouraging to see that other treatment types, (namely photodynamic therapy, antiangiogenic approaches or novel vascular strategies for augumentation of radiation therapy) are capable of inducing changes in tumor vasculature that might also be detectable with PA imaging. As Mallidi and colleagues demonstrate in their recent study, treatment response following photodynamic therapy was strongly correlated with a significant drop in sO_2_ several hours post-treatment [32]. Our current study builds on the findings of that work, as we demonstrate that PA imaging of oxygen saturation is able to predict the therapeutic effect of a burst-release TSL just a few hours post-treatment. We believe that our work, combined with that of others, highlights the considerable potential of PA imaging for the rapid assessment of such treatments at a personalized level.

## Conclusion

This work provides the first example of the use of PA imaging for predicting the therapeutic effect of an ultrafast burst-release TSL treatment, though the study of endogenous sO_2_ values between 30 min to 5 h post-treatment. Our TSL (HaT-DOX) was studied with mild-hyperthermia (HT) and demonstrated a significantly improved therapeutic effect (regression rate of 90%, n = 10), relative to HT-Saline (regression rate: 0%, n = 12). Simulataneously, HT-HaT-DOX and HT-Saline treatments were probed with US-guided PA imaging and a significant drop in sO_2_ (>10%) was observed for every treatment that demonstrated tumor regression by experiment endpoint (i.e. 90% of HT-HaT-DOX treatments). No such drop in sO_2_ was observed for any HT-Saline treatment; nor the single HT-HaT-DOX treatment that showed no regression following treatment. From this data, we suggest a threshold sO_2_ drop can be identified which would be necessary to achieve an effective treatment, and through further investigation, we anticipate this methodology could provide a reliable means for predicting therapeutic outcome within the first few hours of TSL treatment.

## Supporting Information

S1 Fig Schematic representation of the experimental set-ups. (a) The TSL treatment water bath, (b) the US/PA imaging configuration and (c) a schematic of a representative treatment and imaging timecourse with imaging timepoints indicated on the x-axis.
(TIF)

S2 Fig Schematic showing the process for generating tumor sO_2_ maps and histograms. (a) US image of a mouse footpad tumor used for anatomically segmenting the tumor ROI; (b) ROI is applied to the PA images acquired from the 750 nm (top) and 850 nm (bottom) illuminations; (c) The sO_2_ map is reconstructed using the algorithm described in section 2.6; (d) Oxygen saturation histograms were created from the sO_2_ map data for 21 2D slices within a given tumor.
(TIF)

S3 Fig Fluorescent microscopy of tumors treated with HaT-DOX, LTSL-DOX and DOX. Each treatment dosed i.v. (10 mg DOX/kg) and exposed to 1 h of mild-hyperthermia. Following this, tumors were removed, sectioned and nuclei were stained with DAPI. Sections were then studied by fluorescent microscopy to ascertain the relative amounts of DOX present in each tumor.
(TIF)

S4 Fig Body weight plot for the 2 formulations studied. Animals were dosed with either HaT-DOX or Saline and treated with mild-hyperthermia (1 h). Data points are the average of 5 or more animals ± S.D.
(TIF)

S5 Fig Window chamber model of HaT-DOX treatments. During our previous investigations into the HaT-DOX treatment we studied a window chamber model and observed what appeared to be localized hemorrhage and bleeding within the tumor area at 2h following treatment with HT-HaT-DOX. From the timepoints we studied, it appeared that this effect occurred within the first few hours post-treatment. This not only gave us good reason to explore these early timepoints post-treatment, but also suggested that suitable markers for detection of this effect could be something related to the blood–the oxygen saturation of hemoglobin (sO_2_) appeared to be a suitable endogenous marker, which could be studied quantitatively with non-invasive PA imaging.
(TIF)

S6 Fig Representative sO_2_ maps for mice whose endpoint was greater than 7 days. The * denotes the HaT-DOX-treated mouse that did not respond to treatment and whose tumor grew 100% in size. The scale (2 mm) and color bar (0–100%) apply to all images.
(TIF)

S7 Fig Assessments of vessel perfusion. Vessel perfusion for the HT-HaT-DOX treatment is indicated by the white arrows in the magnified image (a), where the overlap of FITC and CD31 appears yellow. Relative number of FITC-perfused vessels following quantification with a Definiens analysis at (b) 2 h and (c) 7 days post-treatment. Significance is represented by * where p < 0.0005.
(TIF)

S1 Table Physical parameters of the HaT-DOX liposomes used in this study. Values are mean ± S.D.
(TIF)
