# Correction: Photoacoustic Imaging of Cancer Treatment Response: Early Detection of Therapeutic Effect from Thermosensitive Liposomes

**DOI:** 10.1371/journal.pone.0174076

**Published:** 2017-03-10

**Authors:** Jonathan P. May, Eno Hysi, Lauren A. Wirtzfeld, Elijus Undzys, Shyh-Dar Li, Michael C. Kolios

The following information is missing from the Funding section: This project was funded in part by the Terry Fox Foundation.

## References

[1] May JP, Hysi E, Wirtzfeld LA, Undzys E, Li S-D, Kolios MC (2016) Photoacoustic Imaging of Cancer Treatment Response: Early Detection of Therapeutic Effect from Thermosensitive Liposomes. PLoS ONE 11(10): e0165345 doi:10.1371/journal.pone.0165345 27788199 10.1371/journal.pone.0165345 PMC5082794

