# Supplementary figures and images for: Photoacoustic Imaging of Cancer Treatment Response: Early Detection of Therapeutic Effect from Thermosensitive Liposomes
Source: PLoS One. 2016 Oct 27;11(10):e0165345. doi: 10.1371/journal.pone.0165345 (PMC5082794; doi:10.1371/journal.pone.0165345)

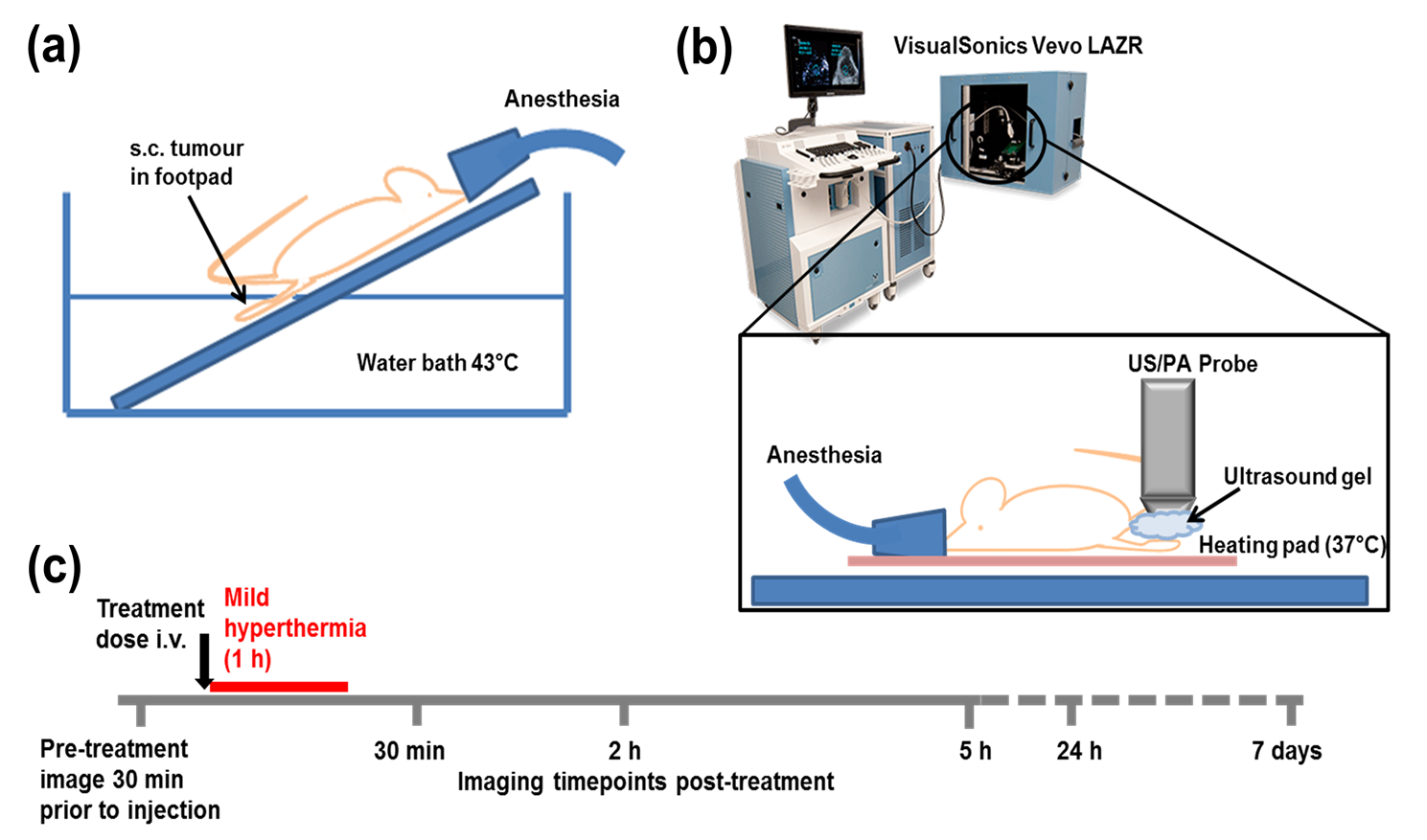

Supplement: S1 Fig — (a) The TSL treatment water bath, (b) the US/PA imaging configuration and (c) a schematic of a representative treatment and imaging timecourse with imaging timepoints indicated on the x-axis. (TIF) [file pone.0165345.s001.tif]

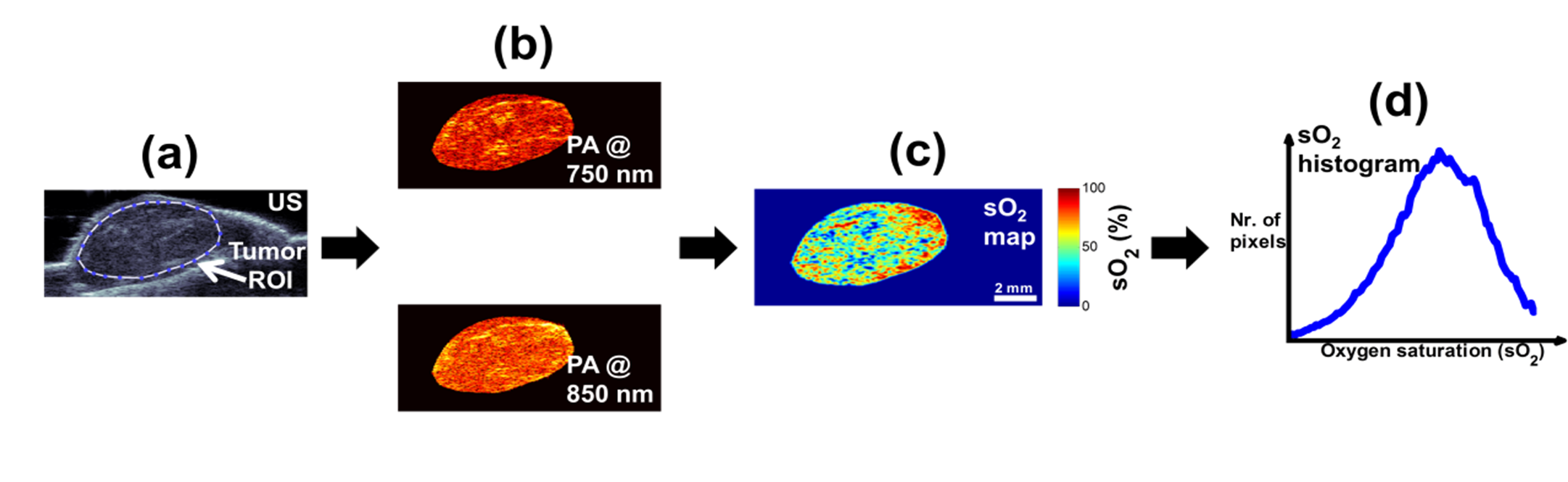

Supplement: S2 Fig — (a) US image of a mouse footpad tumor used for anatomically segmenting the tumor ROI; (b) ROI is applied to the PA images acquired from the 750 nm (top) and 850 nm (bottom) illuminations; (c) The sO2 map is reconstructed using the algorithm described in section 2.6; (d) Oxygen saturation histograms were created from the sO2 map data for 21 2D slices within a given tumor. (TIF) [file pone.0165345.s002.tif]

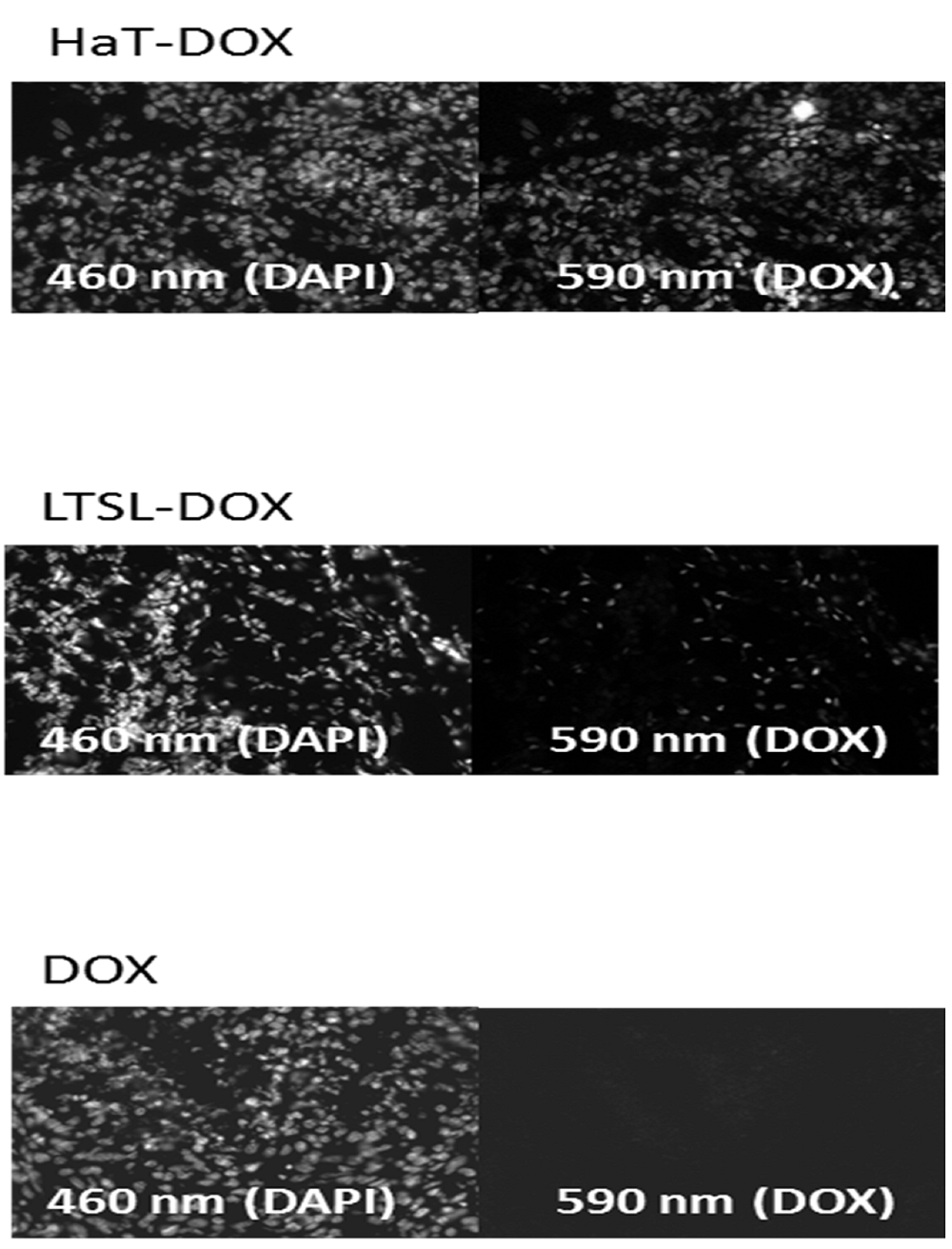

Supplement: S3 Fig — Each treatment dosed i.v. (10 mg DOX/kg) and exposed to 1 h of mild-hyperthermia. Following this, tumors were removed, sectioned and nuclei were stained with DAPI. Sections were then studied by fluorescent microscopy to ascertain the relative amounts of DOX present in each tumor. (TIF) [file pone.0165345.s003.tif]

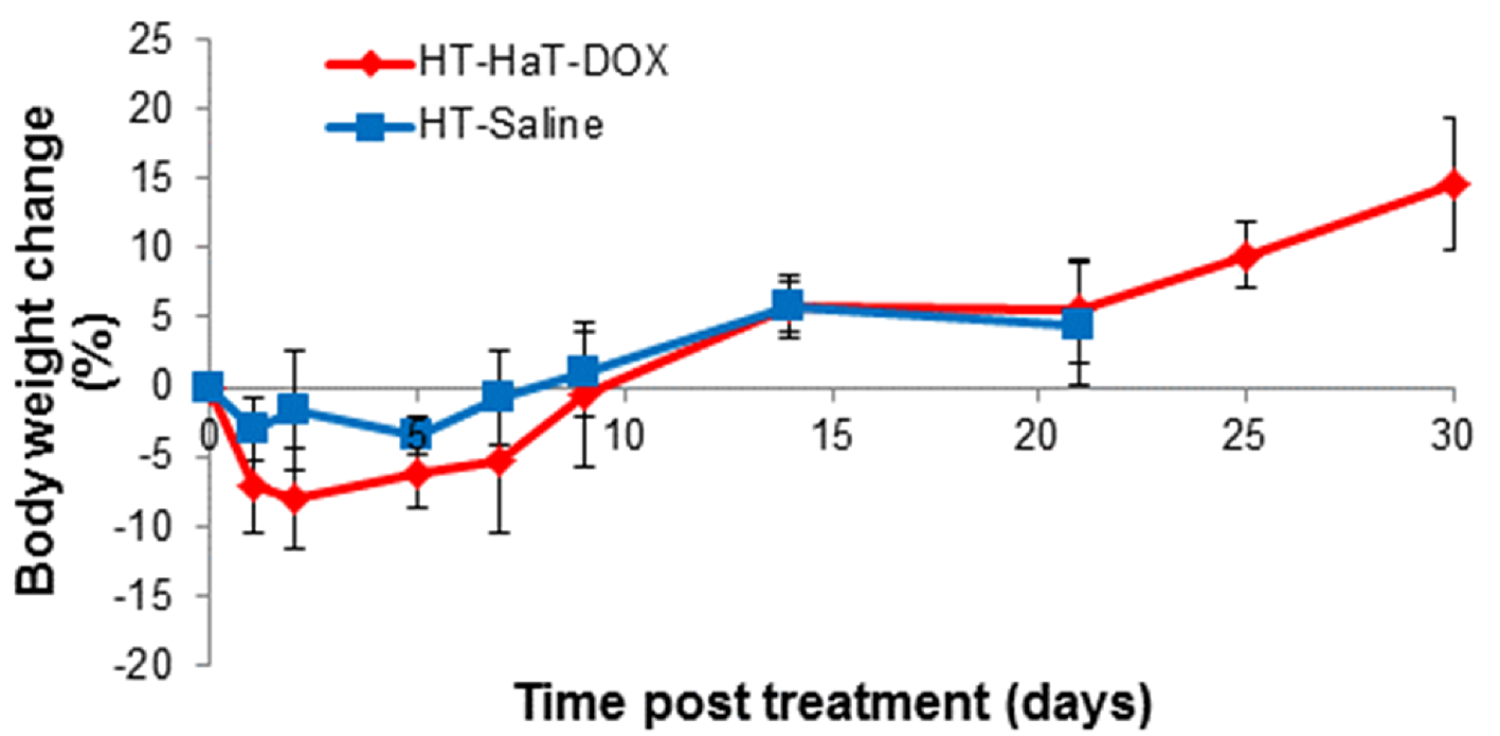

Supplement: S4 Fig — Animals were dosed with either HaT-DOX or Saline and treated with mild-hyperthermia (1 h). Data points are the average of 5 or more animals ± S.D. (TIF) [file pone.0165345.s004.tif]

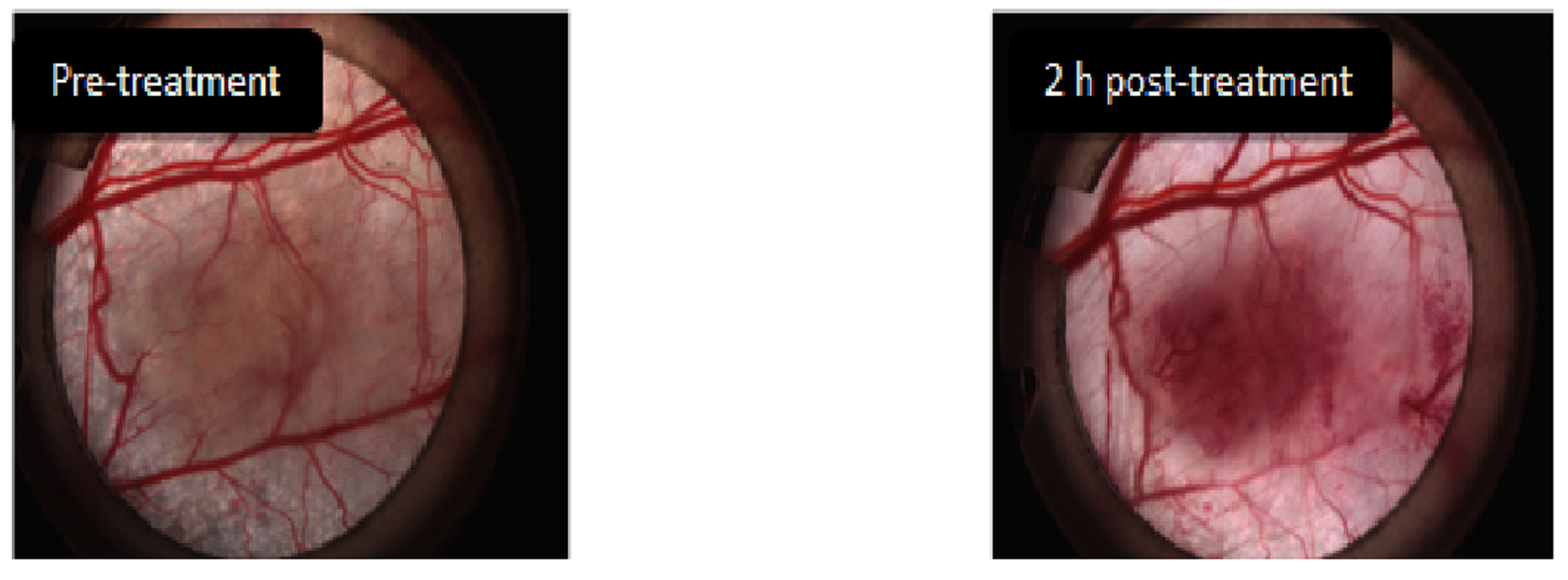

Supplement: S5 Fig — During our previous investigations into the HaT-DOX treatment we studied a window chamber model and observed what appeared to be localized hemorrhage and bleeding within the tumor area at 2h following treatment with HT-HaT-DOX. From the timepoints we studied, it appeared that this effect occurred within the first few hours post-treatment. This not only gave us good reason to explore these early timepoints post-treatment, but also suggested that suitable markers for detection of this effect could be something related to the blood–the oxygen saturation of hemoglobin (sO2) appeared to be a suitable endogenous marker, which could be studied quantitatively with non-invasive PA imaging. (TIF) [file pone.0165345.s005.tif]

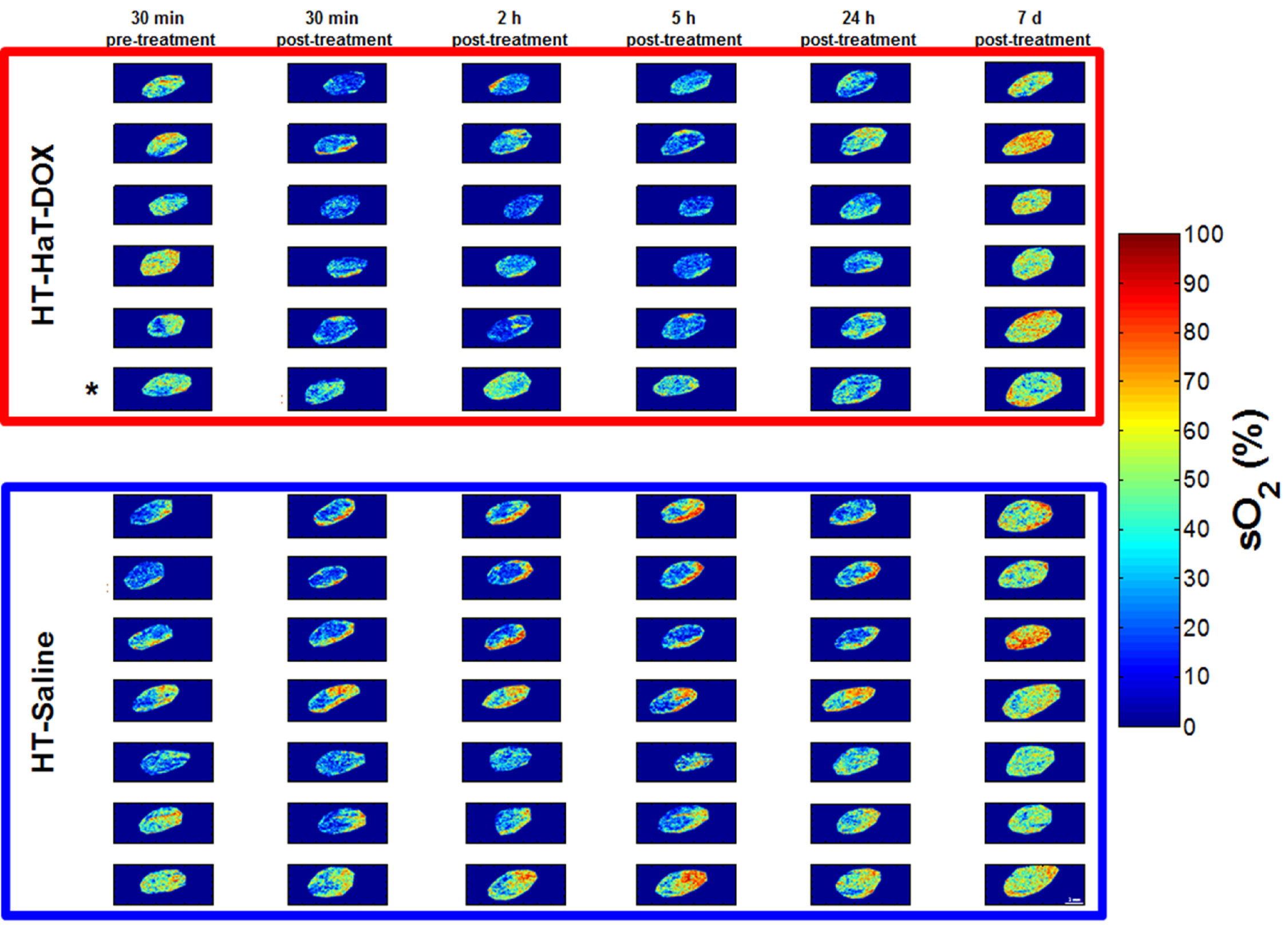

Supplement: S6 Fig — The * denotes the HaT-DOX-treated mouse that did not respond to treatment and whose tumor grew 100% in size. The scale (2 mm) and color bar (0–100%) apply to all images. (TIF) [file pone.0165345.s006.tif]

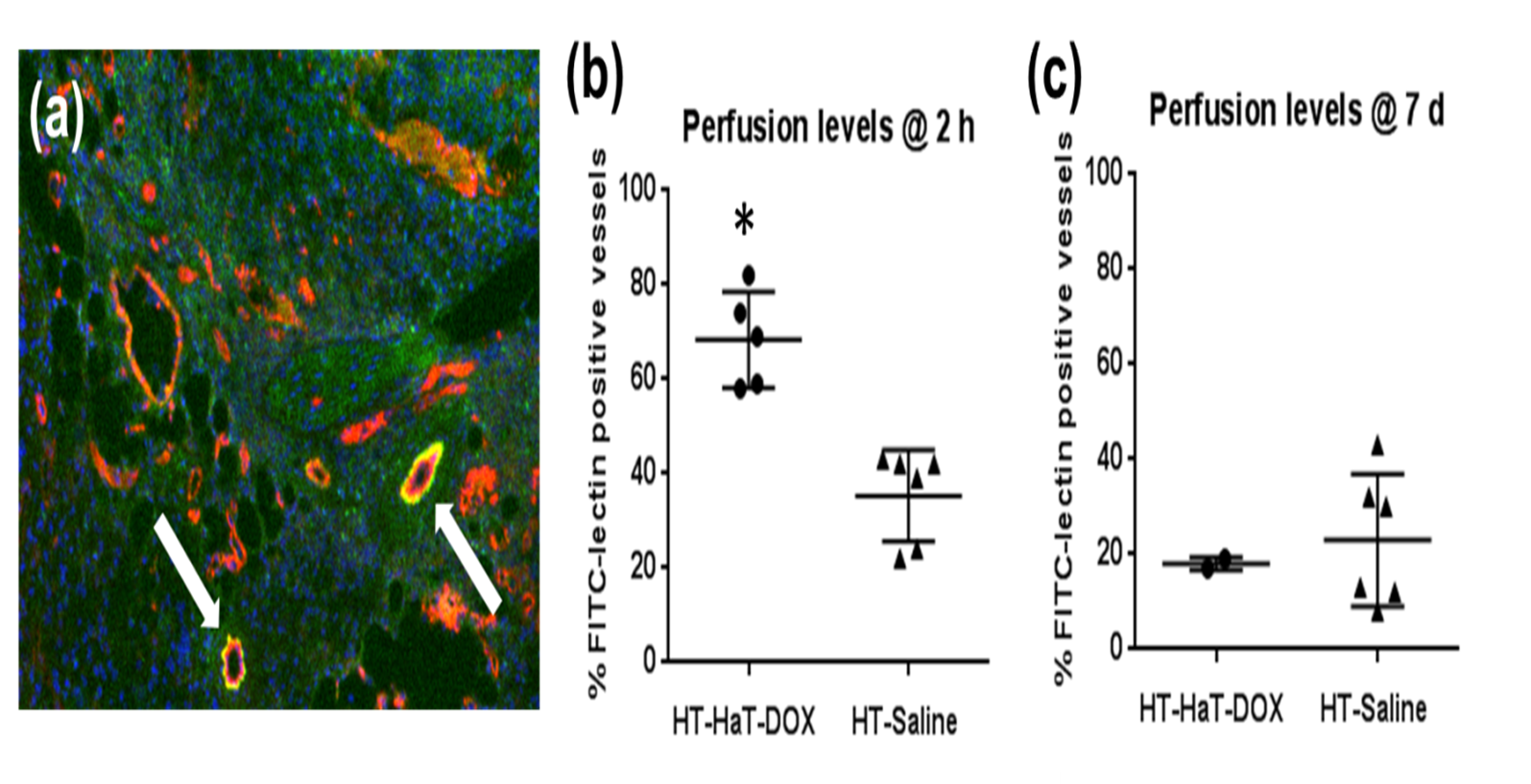

Supplement: S7 Fig — Vessel perfusion for the HT-HaT-DOX treatment is indicated by the white arrows in the magnified image (a), where the overlap of FITC and CD31 appears yellow. Relative number of FITC-perfused vessels following quantification with a Definiens analysis at (b) 2 h and (c) 7 days post-treatment. Significance is represented by * where p < 0.0005. (TIF) [file pone.0165345.s007.tif]

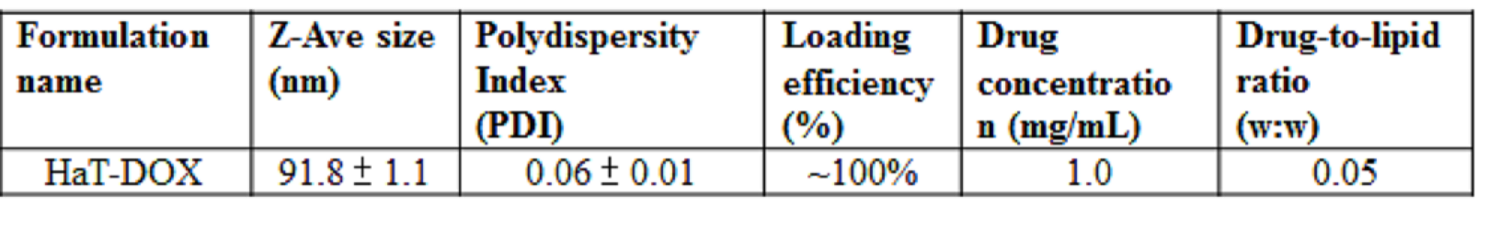

Supplement: S1 Table — Values are mean ± S.D. (TIF) [file pone.0165345.s008.tif]
